# α-Enolase Lies Downstream of mTOR/HIF1α and Promotes Thyroid Carcinoma Progression by Regulating CST1

**DOI:** 10.3389/fcell.2021.670019

**Published:** 2021-04-21

**Authors:** Yang Liu, Lida Liao, Changming An, Xiaolei Wang, Zhengjiang Li, Zhengang Xu, Jie Liu, Shaoyan Liu

**Affiliations:** Department of Head and Neck Surgical Oncology, National Cancer Center/National Clinical Research Center for Cancer/Cancer Hospital, Chinese Academy of Medical Sciences and Peking Union Medical College, Beijing, China

**Keywords:** ENO1, thyroid carcinoma, CST1, mTOR, HIF1α

## Abstract

Novel therapy strategies are crucial for thyroid carcinoma treatment. It is increasingly important to clarify the mechanism of thyroid carcinoma progression. Several studies demonstrate that α-Enolase (ENO1) participates in cancer development; nevertheless, the role of ENO1 in thyroid carcinoma progression remains unclear. In the present study, we found that the expression of ENO1 was upregulated in thyroid carcinoma samples. Proliferation and migration of thyroid carcinoma cells were suppressed by depletion of ENO1; conversely, ENO1 overexpression promoted thyroid carcinoma cell growth and invasion. To elucidate the mechanisms, we found that the hypoxia-related mTOR/HIF1 pathway regulated ENO1 expression. ENO1 regulated the expression of CST1; knockdown of CST1 reversed the tumorigenicity enhanced by ENO1 overexpression. Taken together, our findings provide a theoretical foundation for thyroid carcinoma treatment.

## Introduction

The thyroid gland is the largest endocrine organ in humans; it regulates systemic metabolism (Kondo et al., 2006). Thyroid carcinoma (THCA) is the most frequent malignancy in endocrine organs (Hundahl et al., 1998; Parkin et al., 2005; Kondo et al., 2006; Sipos and Mazzaferri, 2010). More than 85% of thyroid carcinomas arise from follicular epithelial cells, most of which are indolent tumors that can be cured with surgical resection or a combination of radioactive-iodine ablation (Kondo et al., 2006; Paschke et al., 2015). Nevertheless, there is a subset of aggressive thyroid tumors that cannot be treated effectively with current therapies (Kondo et al., 2006). To more efficiently manage thyroid carcinomas, additional effective therapeutic strategies are needed.

A better understanding of thyroid carcinoma means better diagnosis and effective therapy; thus, thyroid carcinoma mortality will further decrease. Over recent decades, the increased incidence of thyroid carcinoma is most likely the result of a better understanding of the genetic pathogenesis and more efficient detection methods (Kondo et al., 2006; Sipos and Mazzaferri, 2010; La Vecchia et al., 2015; Davies, 2016). For example, the incidence increased 311% from 1975 to 2013 in the United States and 150% from 1998 to 2010 in Germany (Paschke et al., 2015; Lim et al., 2017). Meanwhile, thyroid carcinoma mortality has declined (Sipos and Mazzaferri, 2010; La Vecchia et al., 2015; Paschke et al., 2015), primarily due to more sensitive diagnostic tools and more effective therapies, especially rapidly emerging novel targeted strategies (Sipos and Mazzaferri, 2010; La Vecchia et al., 2015). Thus, there is a very high 5-year survival rate (93% for women and 88% for men) for well-differentiated thyroid carcinoma (Paschke et al., 2015).

Clarifying the mechanism of thyroid carcinoma progression is critical for targeted therapy strategy. To date, at least four thyroid carcinoma types have been reported: papillary thyroid carcinoma (PTC), follicular thyroid carcinoma (FTC), anaplastic thyroid carcinoma, and medullary thyroid carcinoma (Sipos and Mazzaferri, 2010; Saiselet et al., 2012). Various mutations causing increased cellular proliferation and dedifferentiation have been characterized for each thyroid carcinoma type (Catalano et al., 2010). *BRAF* point mutations and RET/PTC rearrangements account for 40–60% and 20% of PTC, respectively; these markers can be used to diagnose and treat the most aggressive PTC (Kondo et al., 2006; Xing, 2010). For FTC, the most frequent mutations are RAS point mutations (45% of cases) and PAX8/PPARγ rearrangements (35% of cases). For RAS mutations, MAPK and PI3K pathways could be therapeutic targets (Saavedra et al., 2000; Mitsutake et al., 2005; Knauf et al., 2006). Other mutations have also been described in FTC, including the tumor suppressor gene PTEN (10% of cases) and the PI3KCA oncogenes (10% of cases) (Saiselet et al., 2012). Nevertheless, these are insufficient; more targeted genes and pathways need to be identified.

Cancer cell prefers to metabolize glucose through glycolysis pathway even when oxygen is abundant, a phenomenon called “aerobic glycolysis” or “Warburg effect” (Koppenol et al., 2011). Aerobic glycolysis is driven by activation or upregulation of glycolytic enzymes, such as hexokinase (HK) isoforms, enolase (ENO), and pyruvate kinase (PK). It is well documented that HK2/PKM2 activation of glycolysis contributes to the development of thyroid carcinoma (Coelho et al., 2018). However, the significance of a-Enolase (ENO1) is less unknown in thyroid carcinogenesis. ENO1 is another critical enzyme of the glycolytic pathway expressed in nearly all kinds of tissues (Pancholi, 2001; Zakrzewicz et al., 2014). ENO1 plays critical and multifunctional roles in various physiological and pathological processes (Pancholi, 2001; Ejeskar et al., 2005; Diaz-Ramos et al., 2012; Principe et al., 2015; Capello et al., 2016), including tumorigenesis, cancer invasion, and metastasis (Hsiao et al., 2013; Song et al., 2014). Upregulation of ENO1 was detected in cancers of the stomach, breast, lung, colorectal, brain, kidney, liver, pancreas, and eye, as well as in non-Hodgkin’s lymphoma (Nakajima et al., 1986; Tu et al., 2010; Song et al., 2014; Fu et al., 2015; Liu et al., 2015; Principe et al., 2015; Zhu et al., 2015, 2018; Niccolai et al., 2016; Qian et al., 2017; Zhan et al., 2017). Ectopic ENO1 overexpression promoted tumor formation and tumor chemoresistance (Tu et al., 2010; Song et al., 2014; Fu et al., 2015; Principe et al., 2017; Qian et al., 2017; Zhan et al., 2017; Sun et al., 2019; Chen et al., 2020). Conversely, ENO1 silencing in tumor cells significantly decreased malignant biological behavior (Fu et al., 2015; Principe et al., 2015; Zhao et al., 2015; Capello et al., 2016; Cappello et al., 2017; Huang et al., 2019). Several lines of evidence suggest that ENO1 may be a therapeutic target for endometrial carcinoma (Principe et al., 2015; Zhao et al., 2015; Yin et al., 2018). Nevertheless, the role of ENO1 in human thyroid carcinoma and its putative targets have not been investigated. Therefore, exploring the upstream regulator, the downstream effector and the role of ENO1 in thyroid carcinoma can help the oncologists gain more insights into molecular events during the development of thyroid carcinoma.

mTOR (mechanistic target of rapamycin) is a master regulator of glucose metabolism whose action is mediated by activation of HIF1a (Majumder et al., 2004; Semenza, 2011; Chi, 2012; Cheng et al., 2014; Courtnay et al., 2015; Yang et al., 2015; Xiao et al., 2017). The hypoxia-related mTOR/HIF1α pathway also promotes cell proliferation and metastasis (Xiao et al., 2017; Wei et al., 2018; Pan et al., 2019; Zhang et al., 2019). Nevertheless, it is unclear whether the hypoxia-related mTOR/HIF1α pathway participates in thyroid carcinoma progression. CST1 (cystatin 1), a type 2 cystatin superfamily member, is a specific inhibitor of cysteine proteases (Dai et al., 2017; Oh et al., 2017; Jiang et al., 2018; Liu et al., 2019). Several studies demonstrated that CST1 was closely associated with cell proliferation and metastasis in various cancers, including cancer of the stomach, breast, colorectum, pancreas, and bile duct (Jiang et al., 2018; Cui et al., 2019; Liu et al., 2019; Wang et al., 2020). Wang et al. (2020) predicted that CST1 was a biomarker and therapeutic target for gastric cancer. Nevertheless, the role of CST1 in thyroid carcinoma has not yet been investigated.

In the present study, we demonstrated that ENO1 plays an oncogenic role in thyroid carcinoma progression. First, ENO1 was upregulated in thyroid carcinoma samples. Then, downregulation of ENO1 suppressed cell proliferation, invasion, and *in vivo* tumorigenicity by regulating the cell cycle and apoptosis. ENO1 overexpression promoted proliferation, invasion, and inhibited apoptosis. These findings suggested that ENO1 was an oncogene.

Regarding mechanism, we showed that ENO1 acted downstream of the hypoxia-related mTOR/HIF1a pathway. We demonstrated that ENO1 regulated CST1 expression and that these two proteins acted synergistically in thyroid carcinoma progression. In conclusion, we were the first to identify an oncogenic role of ENO1 in thyroid carcinoma; this will pave the way for a more efficient diagnosis and thyroid carcinoma therapy.

## Materials and Methods

### Patients and Specimens

All clinical tissue samples from patients with PTC and normal adjacent tissues were obtained from populations in China and were collected at the Department of Head and Neck Surgical Oncology, Cancer Hospital, Chinese Academy of Medical Sciences and Peking Union Medical College between 2016 and 2019. The research was approved by the Ethics Committee of National Cancer Center/National Clinical Research Center for Cancer/Cancer Hospital, Chinese Academy of Medical Sciences and Peking Union Medical College and conducted under the guidance of the Declaration of Helsinki. Informed consent regarding the use of specimens was obtained from all patients.

### Cell Culture and Transfection

TPC1 cells and BCPAP cells were cultured in DMEM/F-12 medium (Gibco, Thermo Fisher Scientific) supplemented with 2 mM glutamine, 10% fetal bovine serum (Gibco, Thermo Fisher Scientific), and 100 U/ml penicillin/streptomycin (Sigma, St. Louis, MO, United States). The cells were maintained at 37°C in a humidified atmosphere containing 5% CO_2_. Rapamycin (Sirolimus) was purchased from Selleck (Shanghai, China).

The siRNAs of ENO1 and CST1 were purchased from GenePharma. siCtrl: 5′-UUCUCCGAACGUGUCACGU-3′; siENO1#1: 5′-CGUACCGCUUCCUUAGAACUU-3′; siENO1 #2:5′-GAAUGUCAUCAAGGAGAAAUA-3′; siCST1#1:5′-CC ACCAAAGAUGACUACUA-3′; siCST1#2:5′-GCUCUUUCGAG AUCUACGA-3′; siCST4#1:5′-CUUUCGAGAUCUACGAAGU UCTT-3′; siCST1#2:5′-CCAUCAGCGAGUACAACAATT-3′. According to the manufacturer’s protocol, Lipofectamine RNAiMAX transfection reagent (Invitrogen; Thermo Fisher Scientific) was used for transfection of siRNA. Lipofectamine 3000 (Thermo Fisher Scientific) was used for plasmid overexpression.

### CCK-8 Cell Viability Assay

CCK-8 reagent was purchased from Sigma. Cells seeded into a 96-well plate with a density of 2000 cells per well were maintained for the indicated days, followed by 10 μl CCK-8 reagent added to each well and incubated at 37°C for 3 h. The absorbance at 450 nm was measured using a microplate reader.

### Colony Formation

TPC1 and BCPAP cells were seeded into 6-well plates at 1,000 cells/well, and fresh culture medium was changed every 3 days. Colonies formed after culturing the cells for 14 days. After washing in phosphate-buffered saline (PBS) three times, colonies were fixed in 4% paraformaldehyde for 20 min and stained with GIEMSA staining solution for 20 min. Groups of more than 50 cells were identified as colonies.

### RNA Extraction, Reverse Transcription, and Quantitative Real-Time PCR (qRT-PCR)

TRIzol (Thermo Fisher Scientific) was used for the isolation of total RNAs from cells. The RNAs were reverse-transcribed using M-MLV-RTase (Promega) following the manufacturer’s protocol. The qRT-PCR analysis was performed using SYBR Master Mixture (TAKARA) on the Agilent MX3000p real-time PCR system. qRT-PCR primers were as follows: ENO1 forward, 5′-AAAGCTGGTGCCGTTGAGAA-3′ and reverse, 5′-GGTTGTGGTAAACCTCTGCTC-3′; CST1 forward 5′-GCCTGCGCCAAGAGACA-3′ and reverse, 5′-CCCTGC TGAGCAACAAAGGA-3′; CST4 forward 5′-CCTCTGTGTAC CCTGCTACTC-3′ and reverse, 5′-CTTCGGTGGCCTTG TTGTACT-3′; β-actin forward, 5′-GAGCTGCGTGTGGC TCCC-3′ and reverse, 5′-CCAGAGGCGTACAGGGAT AGCA-3′.

### Apoptosis Analysis

The eBioscience^TM^ Annexin V-FITC apoptosis detection kit (Thermo Fisher Scientific) was used to measure apoptosis. Suspending cells were incubated with 5 μL annexin V-FITC for 10–15 min. After incubation, cells were washed in 1× binding buffer, then resuspended in 1× binding buffer. Resuspended cells were incubated with propidium iodide (20 μg/mL). Finally, samples were subjected to flow cytometry.

### Cell Cycle Analysis

FxCycle PI/RNase Staining Solution (Thermo Fisher Scientific) was used to analyze the cell cycle according to the manufacturer’s protocol. Briefly, cells were trypsinized and centrifuged at 13,000 rpm for 5 min. After washing in iced D-Hanks (pH = 7.2–7.4) buffer, cells were fixed with iced 75% ethanol for at least 1 h. Then, cells were centrifuged and washed in D-Hanks, followed by incubation in 0.5 mL of FxCycle PI/RNase staining solution for 15–30 min at room temperature. Finally, without washing, the analysis was performed using 488-nm excitation and 585/42-nm emission or the equivalent on the Guava easyCyte HT system (Millipore).

### Western Blot Analysis

Cells were lysed with RIPA buffer with proteinase inhibitors. Protein concentrations were measured using the Bradford reagent (Sigma; Merck). Subsequently, 20 μg of total protein was subjected to 10% SDS-PAGE. Then proteins were transferred to nitrocellulose membranes and blocked with 5% non-fat milk at room temperature for 1 h. Membranes were incubated with antibodies against ENO1 (ABclonal, 1:2,000), HIF1a (Sigma, 1:2,000), β-actin (Sigma A5316, 1:5,000), and GAPDH (Proteintech, 1:5,000) at 4°C overnight, followed by secondary antibody (Thermo Fisher Scientific) at room temperature for 60 min.

### Transwell Assay

We seeded 3.0 × 10^4^ cells/well into the upper chamber of 24-well Corning^®^ FluoroBlok TM Cell Culture Inserts (NY, United States) to measure cell migration. The lower chambers were filled with DMEM/F-12 medium supplemented with 10% fetal bovine serum to serve as a chemoattractant. Finally, cells that migrated to the other side of the filter were stained with 0.5% crystal violet and counted under an inverted fluorescence microscope.

### Wound Healing Assay

Cells were plated in 96-well plate and cultured overnight. Wounds were made in confluent monolayer cells using 96 Wounding Replicator (VP Scientific) and cells were cultured in medium supplemented with 1% FBS. Wound healing was detected at 0, 24 h or 0, 48 h for overexpression or knockdown, respectively. The representative fields at different time points were photographed.

### Vector Construction and Luciferase Assay

The TargetScan database^1^ was used to predict potential targets of HIF1a (Jaiswal et al., 2019). The 3′ untranslated region (UTR) of the ENO1 sequence was amplified and cloned into a pGL4.10-report vector (Promega Corporation, Madison, WI, United States). Equal quantities of the pGL4.10-3UTR-ENO1 and Renilla expression vector pRL-TK (Promega Corporation) were co-transfected into 293T cells. The luciferase activity was measured at 48 h post-transfection using the Dual-Glo Luciferase Assay system (Promega Corporation).

### Tumor Xenograft Experiments

We subcutaneously injected 2 × 10^6^ transfected TPC1 cells into the forelegs of immunodeficient nude mice (male BALB/c, 4-week-old). At the indicated times, thyroid tumors were isolated and weighed following international criteria. Animal studies were conducted in compliance with the regulations on management of animal welfare set by the Ethics Committee of National Cancer Center/National Clinical Research Center for Cancer/Cancer Hospital, Chinese Academy of Medical Sciences and Peking Union Medical College. The protocol for animal experiments was approved by the Ethics Committee of National Cancer Center/National Clinical Research Center for Cancer/Cancer Hospital, Chinese Academy of Medical Sciences and Peking Union Medical College.

### RNA Sequencing

Total RNA was extracted from TPC1 cells incubated with exosomes using TRIzol reagent (Invitrogen, Carlsbad, CA, United States). Agarose electrophoresis was used to determine the total RNA’s integrity, and a NanoDrop (Thermo Scientific NanoDrop 2000 Microvolume Spectrophotometer, RRID:SCR_018042) was used for quality control and quantification. A sequencing library was constructed using an RNA library construction kit (NEB, United States). Their sequences were analyzed using Illumina HiSeq^TM^ 2000 (Wistar Genomics Facility, RRID:SCR_010205; Illumina Inc., San Diego, CA, United States).

### Chromatin Immunoprecipitation (Ch-IP)

TPC1 cells were fixed in 1% formaldehyde for 10 min at 4°C. Cells were then sonicated to shear genomic DNA, followed by incubating overnight with 5 μg of Rb IgG or HIF1α antibody (Sigma, 1:2,000). The resulting complexes were precipitated using Fastflow G-Sepharose beads (GE Healthcare, Little Chalfont, United Kingdom), eluted, purified, and analyzed using PCR.

### Gene-Set-Enrichment Analysis (GSEA)

According to their *P*-values, a GSEA algorithm was used to identify the enrichment of specific functions in the list of genes pre-ranked to test differences in expression between cell lines. The statistical significance of the enrichment score was calculated by permuting the genes 1,000 times as performed by the GSEA software. To collapse each probe set on the array to a single gene, we selected the probe with the highest variance among multiple probes corresponding to the same gene. Gene sets were derived from the REACTOME pathway database.

### Bioinformatics Analysis

The StarBase V3.0 project^2^ was used to analyze expression levels of ENO1 and CST1. Correlation between ENO1 and CST1 was obtained from 510 samples in thyroid carcinoma.

### Statistical Analysis

The analysis was performed using GraphPad Prism 6.0 software. Differences were analyzed using the Student’s *t*-test or one-way ANOVA. Differences were statistically significant when *P* < 0.05.

## Results

### Upregulation of ENO1 in Thyroid Carcinoma

**FIGURE 1:**
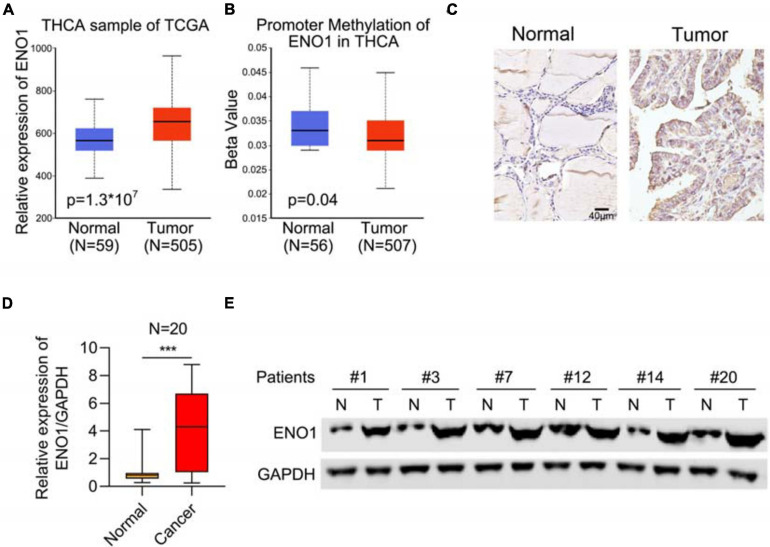
Upregulation of ENO1 in thyroid carcinoma. **(A)** Analysis of ENO1 expression in thyroid carcinoma (*n* = 505) and normal samples (*n* = 59) from THCA. **(B)** Analysis of promoter methylation of ENO1 in thyroid carcinoma (*n* = 507) and normal samples (*n* = 56) from THCA. **(C)** IHC analysis of ENO1 expression in normal and thyroid carcinoma tissues. **(D)** qRT-PCR analysis of ENO1 expression in normal and thyroid carcinoma tissues. **(E)** Western blotting analysis of ENO1 expression in normal and thyroid carcinoma tissues. GAPDH serves as a loading control. ***p < 0.001.

To study the function of ENO1 in thyroid carcinoma, we first analyzed the THCA database to determine the relevance of ENO1 in thyroid carcinoma. We found upregulation of ENO1 in thyroid carcinoma compared with normal samples (Figure 1A). And we also found that the higher level of methylation of ENO1’s promoter in normal group than tumor group (Figure 1B). IHC staining showed that ENO1 was upregulated in thyroid carcinoma samples as compared with normal samples (Figure 1C and Table 1). The expression ENO1 was not associated with age, gender, T classification and lymph node metastasis (Table 2). qRT-PCR and western blotting were also performed to confirm these results; there were higher ENO1 mRNA and protein levels in thyroid carcinoma samples (Figures 1D,E). These findings suggest that ENO1 might be an oncogene.

**TABLE 1:** The expression of ENO1 in Normal tissue and thyroid carcinoma by IHC.

	**Normal**	**Tumor**	**χ^2^**	***P* Value**
ENO1 high expression	17	31	8.75	0.003
ENO1 low expression	28	14		
Total	45	45		

**TABLE 2:** Correlation of ENO1 expression with clinicopathological characteristics in 45 patients of thyroid carcinoma.

	**Characteristic**	**ENO1 expression**		***P* value**
		**High**	**Low**	
Age	<55	14	12	0.787
	≥55	11	8	
Gender	Male	5	6	0.803
	Female	14	20	
T classification	T1–T2	6	8	0.457
	T3–T4	17	14	
Lymph node metastasis	Yes	20	9	0.098
	No	7	9	

### Downregulation of ENO1 Suppresses Cell Proliferation, Invasion, and *in vivo* Tumorigenicity

**FIGURE 2:**
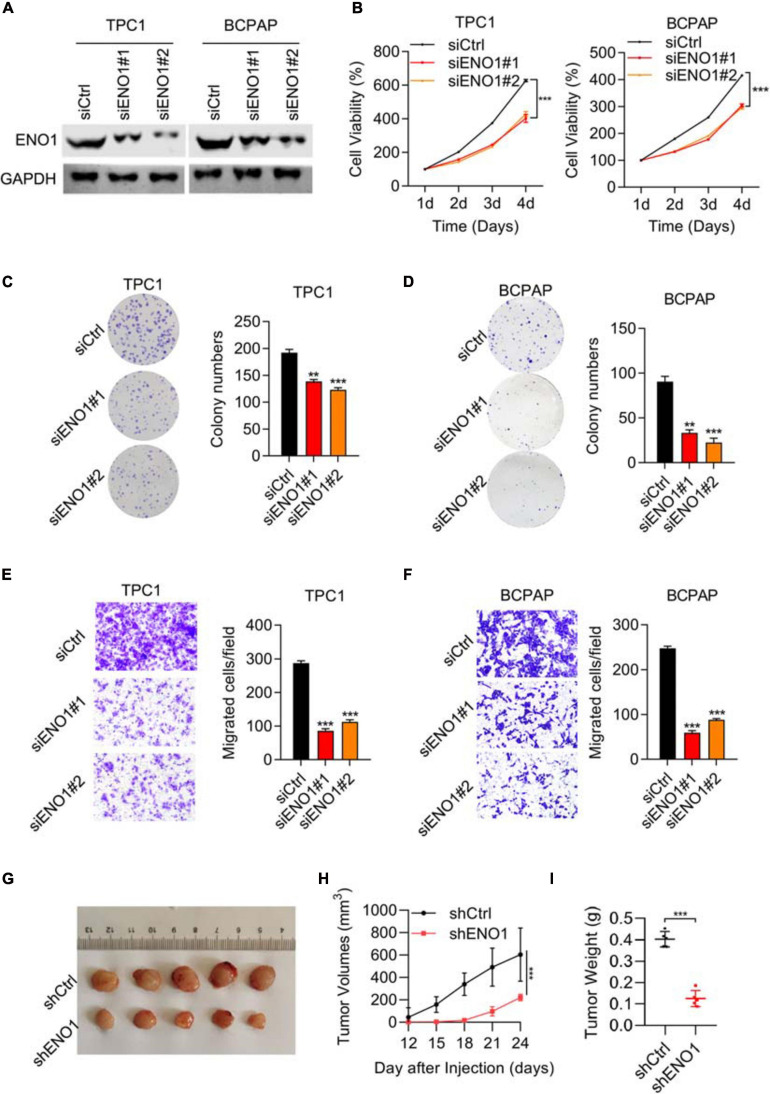
Downregulation of ENO1 suppresses cell proliferation, invasion, and *in vivo* tumorigenicity. **(A)** Western blotting analysis of ENO1 knockdown efficiency in TPC1 and BCPAP cells. GAPDH was used as a loading control. **(B)** CCK8 analysis of cell proliferation in TPC1 cells and BCPAP cells transfected with siCtrl, siENO1#1, and siENO1#2. **(C,D)** Colony formation assay in TPC1 **(C)** and BCPAP **(D)** cells transfected with siCtrl, siENO1#1, and siENO1#2. *P < 0.05, **P < 0.01, ***P < 0.001. **(E,F)** Transwell invasion analysis in TPC1 **(E)** and BCPAP **(F)** cells transfected with siCtrl, siENO1#1, and siENO1#2. ***P < 0.001. **(G–I)** Athymic nude mice (*n* = 5) injected with TPC1 cell lines expressing shCtrl or shENO1 were analyzed for tumor formation. Representative image **(G)**, tumor growth curve **(H)** and weight **(I)** of xenografted tumors are shown.

To elucidate the role of ENO1 in thyroid carcinoma progression, small interfering RNAs were identified to downregulate ENO1 expression in TPC1 and BCPAP cells. Western blotting showed high knockdown efficiency of siENO1 in both cell lines (Figure 2A). Then, a CCK8 assay was performed to measure cell proliferation. Depletion of ENO1 suppressed TPC1 and BCPAP thyroid carcinoma cells’ proliferation to nearly half that of control cells (Figure 2B). The colony formation assay demonstrated that fewer colonies were formed in siENO1 cells (Figures 2C,D).

Regarding migration ability, transwell results showed that the percentage of migration cells was decreased (greater than 50% in both cells) with ENO1 depletion compared to control cells (Figures 2E,F). In addition, wound healing assays demonstrated that ENO1 knockdown suppressed the migration capacity of TPC1 and BCPAP cells (Supplementary Figure 1A). To explore the function of ENO1 *in vivo*, a tumor formation assay was performed. We used ENO1 shRNA to downregulate the expression of ENO1, and the tumor was isolated for measurements at indicated times. Isolated tumors only weighed 0.1 g in shENO1 samples compared with 0.4 g in the control samples (Figures 2G–I). These findings further suggest an oncogenic role for ENO1.

### ENO1 Regulates the Cell Cycle and Apoptosis

**FIGURE 3:**
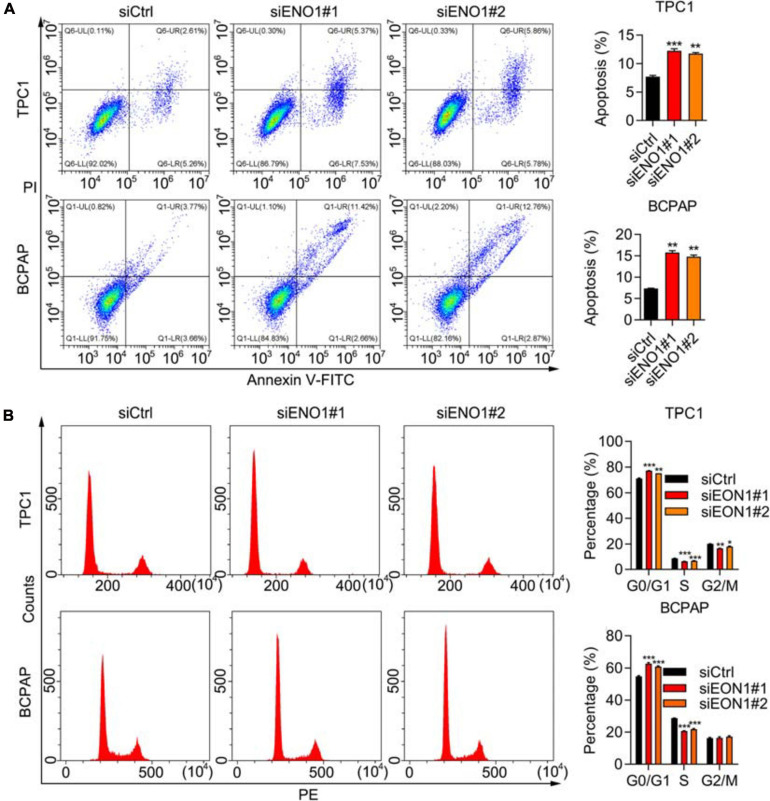
ENO1 regulates cell cycle and apoptosis. **(A)** Cell cycle analysis using PI staining and flow cytometry in siCtrl, siENO1#1, and siENO1#2 transfected into TPC1 and BCPAP cells. **P < 0.01, ***P < 0.001. **(B)** Flow cytometry analysis of apoptosis using annexin V-FITC and PI staining in TPC1 and BCPAP cells transfected with siCtrl, siENO1#1, and siENO1#2. ***P < 0.001, **P < 0.01, *p < 0.05.

Because the cell cycle is frequently disturbed by oncogenes, we performed flow cytometry. The depletion of ENO1 decreased the number of cells in the S phase, while the G0/G1 cells’ percentage increased (Figure 3A). Apoptotic cells significantly increased with ENO1 depletion. Compared with control cells, the percentage of apoptosis nearly doubled in siENO1 cells in bPC1 and BCPAP cells (Figure 3B). These findings suggest that cell cycle progression was severely impaired by ENO1 depletion.

### ENO1 Overexpression Promotes Proliferation and Invasion and Inhibits Apoptosis

**FIGURE 4:**
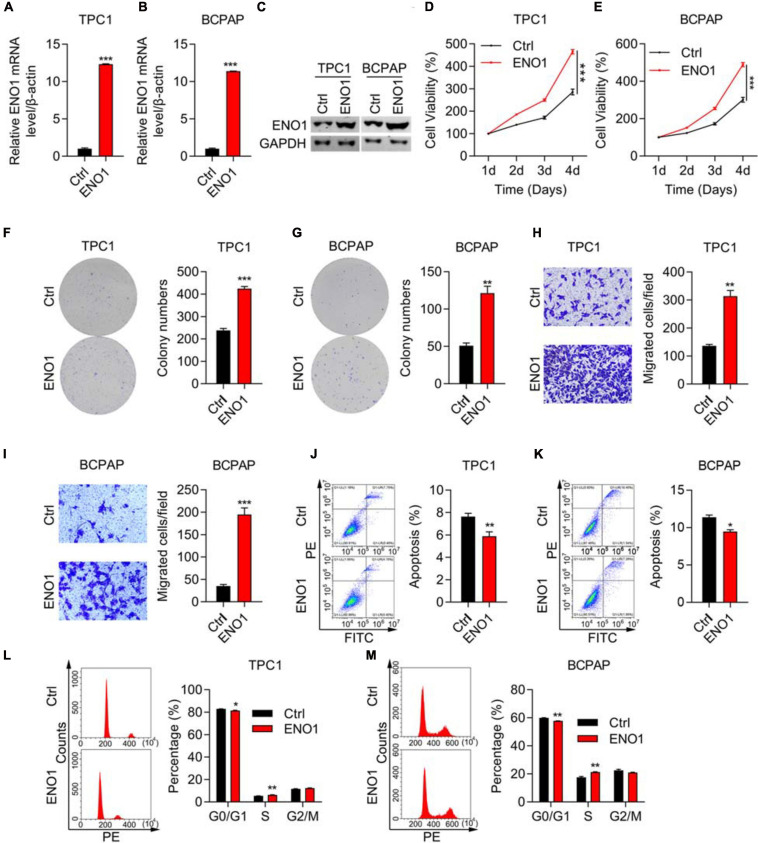
ENO1 overexpression promotes proliferation and invasion and inhibits apoptosis. **(A,B)** qRT-PCR analysis of ENO1 overexpression in TPC1 **(A)** and BCPAP **(B)** cells. β-actin was used for normalization, ***P < 0.001. **(C)** Western blotting analysis of ENO1 overexpression in TPC1 and BCPAP cells. GAPDH was used as a loading control. **(D,E)** CCK8 cell viability analysis of control or ENO1-overexpressed TPC1 **(D)** and BCPAP **(E)** cells, ***P < 0.001. **(F,G)** Colony formation assay of control or ENO1-overexpressed TPC1 **(F)** and BCPAP **(G)** cells. **P < 0.01, ***P < 0.001. **(H,I)** Transwell invasion analysis of control or ENO1-overexpressed TPC1 **(H)** and BCPAP **(I)** cells. ***P < 0.001, **P < 0.01. **(J,K)** Flow cytometry analysis of apoptosis using Annexin V-FITC and PI staining in control or ENO1-overexpressed TPC1 **(J)** and BCPAP **(K)** cells. *P < 0.05, **P < 0.01. **(L,M)** Cell cycle was measured using PI staining and flow cytometry in control or ENO1-overexpressed TPC1 **(L)** and BCPAP **(M)** cells, *P < 0.05, **P < 0.01.

To confirm the knockdown results, we performed overexpression experiments. We performed qRT-PCR and western blotting to demonstrate high overexpression levels of ENO1 in both TPC1 and BCPAP cells (Figures 4A–C). Next, the CCK8 assay demonstrated that ENO1 overexpression significantly improved cell viability compared with control cells from 1 to 4 days (Figures 4D,E). The ability of colony formation (Figures 4F,G) and Transwell migration (Figures 4H,I) in ENO1 overexpressing cells were consistent with the knockdown results (Figures 2D–F). Wound healing results also showed that ENO1 overexpression enhanced the migration capacity of TPC1 and BCPAP cells (Supplementary Figure 1B). We also performed flow cytometry analysis. Apoptosis was inhibited with ENO1 overexpression in both TPC1 and BCPCP cells (Figures 4J,K). Never the less, ENO1 accelerated cell cycle progression, with S stage cells increasing and G0/G1 stage cells decreasing (Figures 4L,M). These findings suggest that ENO1 regulates the cell cycle and apoptosis to promote cancer cell progression, functioning as an oncogene.

### mTOR/HIF1α Regulates Expression of ENO1

**FIGURE 5:**
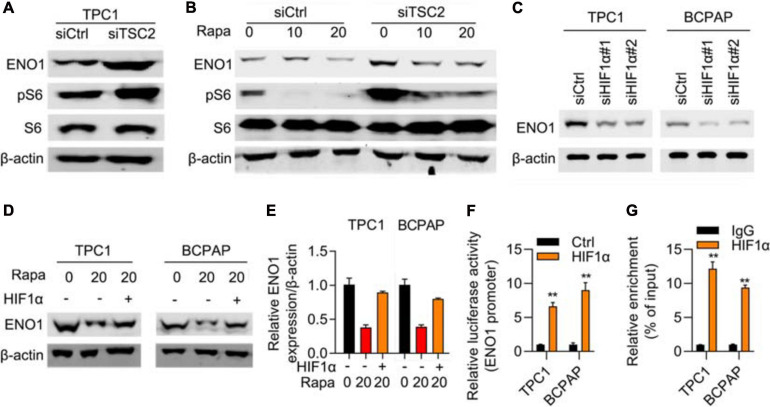
mTOR/HIF1α regulates expression of ENO1. **(A)** ENO1 and phosphorylated S6 was upregulated in TSC1 knockout TPC1 cells. β-actin was used as a loading control. **(B)** ENO1 was downregulated with rapamycin treatment. β-actin was used as a loading control. **(C)** Depletion of HIF1α downregulated expression of ENO1. β-actin was used as a loading control. **(D–E)** HIF1α rescued downregulation of ENO1 with rapamycin treatment. Western blotting **(D)** and qRT-PCR **(E)** were shown. **P < 0.01. **(F)** ENO1 luciferase assay in control and HIF1α transfected cells. **P < 0.01. **(G)** HIF1α targeted the promoter region of ENO1. ChIP-qPCR results are shown. **P < 0.01.

Next, we elucidated the mechanism of ENO1’s oncogenic role. ENO1 is a glycolysis-associated enzyme that participates in various cancers (Nakajima et al., 1986; Tu et al., 2010; Song et al., 2014; Fu et al., 2015; Liu et al., 2015; Principe et al., 2015; Zhu et al., 2015, 2018; Niccolai et al., 2016; Qian et al., 2017; Zhan et al., 2017); therefore, we focused on glycolysis-associated pathways; the mTOR/HIF1α pathway is also involved in glycolysis (Majumder et al., 2004; Semenza, 2011; Chi, 2012; Cheng et al., 2014; Courtnay et al., 2015; Yang et al., 2015; Xiao et al., 2017). Therefore, we examined ENO1 expression in TSC1 knockout MEF cells and found that TSC1 depletion aberrantly activated the mTOR pathway (Cao et al., 2020). Western blotting showed that knockout of TSC1 upregulated ENO1 expression and the phosphorylation of S6, the downstream of mTOR pathway (Figure 5A), as did HIF1α, another downstream molecule of the mTOR/HIF1α pathway. To confirm these results, we used rapamycin to inhibit the mTOR pathway (Edwards and Wandless, 2007). ENO1 was downregulated upon rapamycin addition, more significantly in TSC1-depleted cells (Figure 5B).

To explore the relationship between ENO1 and HIF1α, an RNA interference assay was performed. With HIF1α depletion, ENO1 was downregulated in both TPC1 and BCPAP cells (Figure 5C). The addition of rapamycin further reduced ENO1 expression. Overexpression of HIF1α rescued ENO1 expression both at the protein and mRNA levels (Figure 5D). We also performed a luciferase assay. The luciferase activity increased in HIF1α-transfected cells compared with control cells (Figure 5E). The ChIP-qPCR assay demonstrated that the *HIF1*α targeted the promoter region of *ENO1* (Figures 5F,G). These findings suggest that ENO1 is located downstream of and is regulated by the mTOR/HIF1α pathway.

### ENO1 Regulates the Expression of CST1 and CST4

**FIGURE 6:**
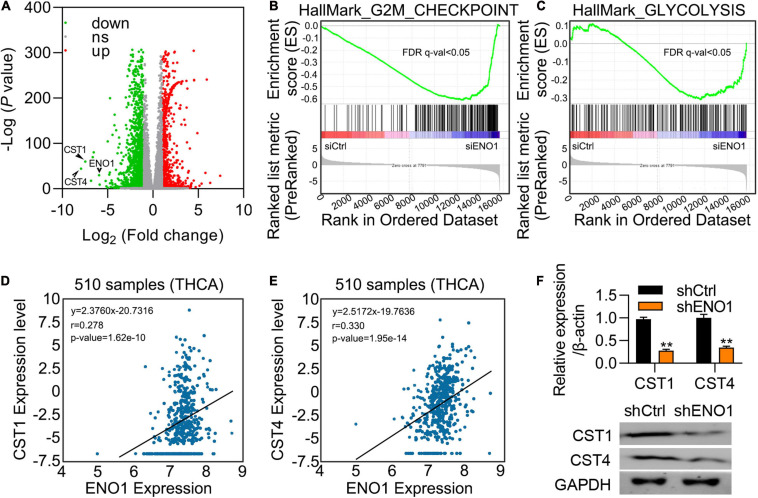
ENO1 regulates the expression of CST1. **(A)** Differential expression of mRNAs between control and siENO1 cells. The abscissa was log2, and the ordinate was -log10. The red and green dots represent upregulated and downregulated genes, respectively. The gray dots represent genes that were not differentially expressed between the two groups. **(B,C)** Selected enriched gene sets from the GSEA. The green tracing shows the enrichment score based on hits of genes in the ordered list of differentially regulated genes resulting from the comparison of control and ENO1 depletion cells. **(D,E)** Correlation between ENO1 and CST1 **(D)** and CST4 **(E)** expression (510 samples) in TCGA database. **(F)** Downregulation of CST1 and CST4 expression by ENO1 depletion. qRT-PCR are shown. β-actin was used for normalization. **P < 0.01.

To further explore the mechanism, we performed differential expression analysis between ENO1-depleted cells and control cells. Variations of mRNA expression between the two groups were plotted, and CST1 and CST4 are the most decreased genes by ENO1 knockdown (Figure 6A). GESA analysis showed that ENO1 depletion altered downregulated including G2M checkpoints and glycolysis signaling pathways (Figure 6B). We chose the genes with a maximum difference for further analysis (Figure 6C). Correlation analysis of the THCA database showed that CST1 and CST4 expression were positively correlated with ENO1 expression (Figures 6D,E). qRT-PCR and immunoblotting confirmed that depletion of ENO1 downregulated CST1 and CST4 expression at the mRNA and protein levels (Figure 6F).

### Depletion of CST1, but Not CST4, Inhibits Proliferation and Invasion and Promotes Apoptosis

**FIGURE 7:**
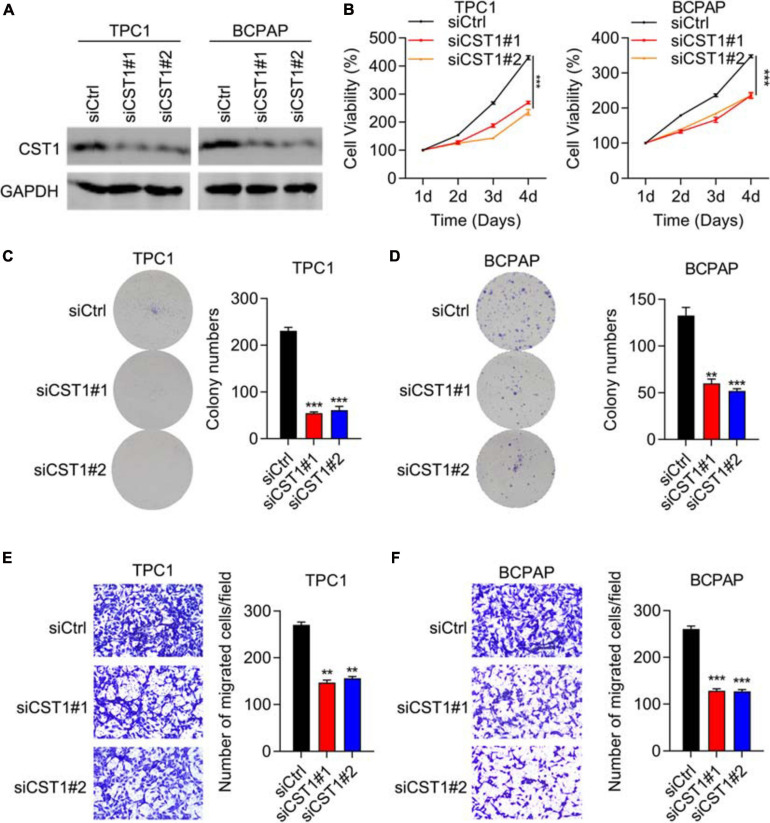
Depletion of CST1 inhibits proliferation and invasion and promotes apoptosis. **(A)** Effective knockdown of CST1 in TPC1 and BCPAP cells. Western blot of TPC1 and BCPAP cells transfected with siCtrl, siCST1#1, and siCST1#2. GAPDH served as a loading control. **(B)** Cell viability analysis of siCtrl, siCST1#1, and siCST1#2 transfected into TPC1 and BCPAP cells using the CCK8 assay. ***P < 0.001. **(C,D)** Colony formation analysis of siCtrl, siCST1#1, and siCST1#2 transfected into TPC1 **(C)** and BCPAP **(D)** cells. **P < 0.01; ***P < 0.001. **(E,F)** Invasion analysis of siCtrl, siCST1#1, and siCST1#2 transfected into TPC1 **(E)** and BCPAP **(F)** cells. **P < 0.01; ***P < 0.001.

CST1 and CST4 were previously reported to be oncogenes (Jiang et al., 2018; Cui et al., 2019; Liu et al., 2019; Wang et al., 2020). Next, we studied the role of CST1 and CST4 in thyroid carcinoma. Western blotting confirmed the high knockdown efficiency of siCST1 in both TPC1 and BCPAP cells (Figure 7A). Cell viability analysis with CCK8 assay showed that depletion of CST1 significantly reduced cell viability (Figure 7B). However, we found that knockdown of CST4 could not affect the proliferation of THCA cells (Supplementary Figure 2). Colony numbers also decreased in siCST1 cells (Figures 7C,D). Invasion ability was also inhibited with CST1 depletion, decreasing to nearly half of the control cell levels (Figures 7E,F). These findings suggest that CST1 is an oncogene in thyroid carcinoma.

### Proliferation, Invasion, and Tumorigenicity Enhanced by ENO1 Overexpression Is Attenuated by CST1 Depletion

**FIGURE 8:**
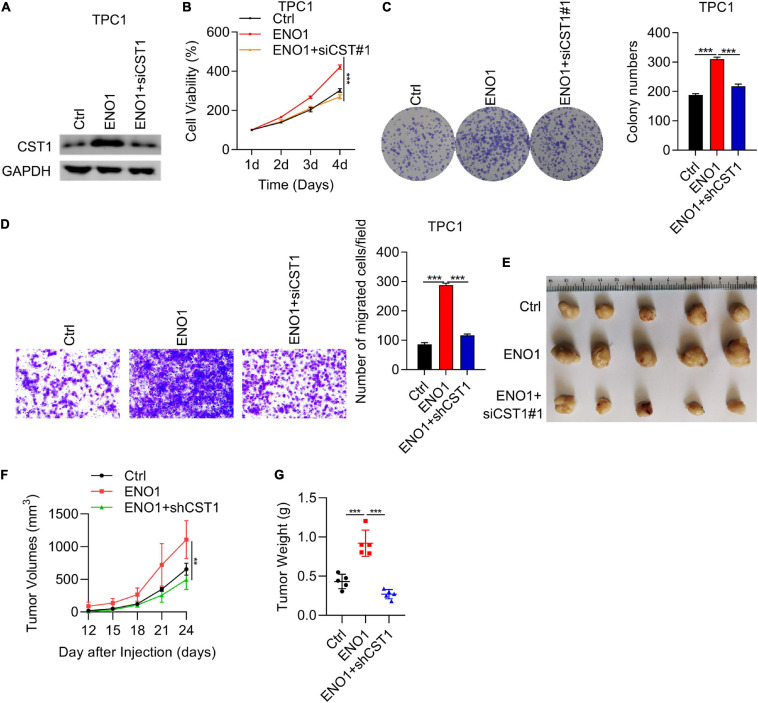
Proliferation, invasion, and tumorigenicity enhanced by ENO1 overexpression is attenuated by CST1 depletion. **(A)** Western blot analysis of CST1 in TPC1 cells transfected with control, ENO1, and ENO1+shCST1. GAPDH was used as a loading control. **(B)** CCK8 cell viability analysis of TPC1 cells transfected with control, ENO1, and ENO1+shCST1. ***P < 0.001. **(C)** Colony formation analysis of TPC1 cells transfected with control, ENO1, and ENO1+shCST1. ***P < 0.001. **(D)** Invasion ability analysis of TPC1 cells transfected with control, ENO1, and ENO1+shCST1. ***P < 0.001. **(E–G)** Tumor formation analysis in athymic nude mice (*n* = 5) injected with TPC1 cell lines expressing control, ENO1, and ENO1+shCST1. Representative image **(E)**, tumor growth curve **(F)** and tumor **(G)** weight of xenografted tumors are shown. **P < 0.01; ***P < 0.001.

To further clarify the relationship between ENO1 and CST1 in thyroid carcinoma, we combined overexpression and knockdown assays in TPC1 cells. Western blotting showed that ENO1 overexpression upregulated CST1 expression and reduced it after shCST1 treatment (Figure 8A). Next, cell viability and colony formation assays were performed. Knockdown of CST1 significantly reduced cell viability enhanced with ENO1 overexpression to control level (Figures 8B,C). Invasion ability was also attenuated by CST1 depletion (Figure 8D). Finally, *in vivo* tumorigenicity was measured. Following the previous result (Figure 2G), overexpression of ENO1 resulted in larger tumor sizes (Figures 8E–G), suggesting that ENO1 was an oncogene. As predicted, tumor size enlargement was reduced to control levels with shCST1 treatment (Figures 8E–G). These findings suggest a synergistic role between ENO1 and CST1 in thyroid carcinoma progression.

## Discussion

Traditional therapy strategies such as radioactive-iodine ablation and surgical resection are essential for thyroid carcinoma treatment. Currently, novel targeted strategies play increasingly essential roles in thyroid carcinoma treatment, especially for aggressive thyroid tumors (Kondo et al., 2006). Therefore, more potential targeted genes need to be identified, and their mechanisms need to be further clarified. Our study demonstrated that ENO1 was an oncogene in thyroid carcinoma that acted downstream of mTOR/HIF1α and accelerated thyroid carcinoma progression via regulating CST1. Because ENO1 has been identified as an oncogene in several cancers (Nakajima et al., 1986; Tu et al., 2010; Song et al., 2014; Fu et al., 2015; Liu et al., 2015; Principe et al., 2015; Zhu et al., 2015, 2018; Niccolai et al., 2016; Qian et al., 2017; Zhan et al., 2017), it could be a potential novel target for thyroid carcinoma. Our study paves the way for targeted strategies of thyroid carcinoma treatment.

Enolase has three isoforms in higher eukaryotes: ENO1 is widely expressed in nearly all tissues, whereas ENO2 and ENO3 are specifically expressed in neurons and muscles, respectively (Chen et al., 2020). Although there have been several reports of ENO1, the functions of ENO2 and ENO3 have been rarely reported. Whether ENO2 and ENO3 function as oncogenes in neurons and muscles needs to be clarified. We also need to understand whether the functions of the three ENOs are synergistic or antagonistic.

The localization of ENO1 varies depending on its function. In the cytoplasm, ENO1 maintains ATP levels and associates with the cytoskeleton. At the cell membrane, ENO1 activates the plasminogen system to accelerate tumor cell invasion. ENO1 also localizes in the nucleus to regulate c-myc expression and impede tumor growth (Cappello et al., 2017; Yin et al., 2018). To further explore the mechanism of ENO1 as an oncogene, the precise localization of ENO1 in thyroid carcinoma cells needs to be identified.

The Warburg effect occurs in most tumor cells (Warburg et al., 1927). In these cells, glycolysis is enhanced. The tricarboxylic acid cycle is inhibited even in abundant oxygen because enhanced glycolysis can provide more efficient glucose consumption to promote tumor cell growth (Lunt and Vander Heiden, 2011). As a glycolytic enzyme, ENO1 overexpression enhances the glycolytic pathway (Yin et al., 2018). There are also reports that PI3K/AKT/mTOR/HIF1α upregulates the expression of glucose transporters and glycolytic enzymes (Majumder et al., 2004; Semenza, 2011; Chi, 2012; Cheng et al., 2014; Courtnay et al., 2015; Yang et al., 2015; Xiao et al., 2017); HIF1α also targets ENO1 (Qian et al., 2017). Therefore, enhanced glycolysis may be the reason why mTOR/HIF1α/ENO1 promotes thyroid carcinoma progression. The mTOR/HIF1α/ENO1 pathway can also enhance glycolysis in other cancers. However, all these effects need to be further clarified.

Type 2 cystatin superfamily, consisting of CST1, CST2, CST3, CST4, and CST5, exerts cysteine proteases inhibitor effect and is widely expressed in multiple organs. Increasing evidences have demonstrated that dysregulation of CST1 contributes to the development of various cancers. For instance, CST1 expression was elevated in and conferred poor prognosis of breast cancer patients. CST1 also promoted the malignant function of breast cancer cells (Dai et al., 2017). Overexpression of CST1 also contributed colorectal cancer growth via reducing intracellular reactive oxygen species (ROS) production (Oh et al., 2017). In addition, upregulation of CST1 promoted hepatocellular carcinogenesis and predicted worse prognosis for the patients (Cui et al., 2019). Besides, elevation of CST1 is a promising diagnostic biomarker for other cancer types, including pancreatic cancer (Jiang et al., 2015), esophageal squamous cell carcinoma (Chen et al., 2013), and gastric cancer (Choi et al., 2009). Nevertheless, the significance of CST1 in thyroid carcinoma and the upstream regulator of CST1 remain to be determined. In this study, we presented the data that ENO1 positively regulated the expression of CST1 and CST4 in thyroid carcinoma cells. There was a positive correlation between ENO1 and CST1/CST4 in human thyroid carcinoma tissues. Functional experiments demonstrated that knockdown of CST1, but not CST4, suppressed the growth of growth of thyroid carcinoma cells. Furthermore, knockdown of CST1 reduced the migration capacity of thyroid carcinoma cells. Importantly, CST1 silencing reversed the oncogenic function of ENO1 on thyroid carcinoma cell growth, migration and tumorigenic capacity. These results suggested that ENO1 upregulation of CST1 contributed to thyroid carcinoma growth, migration and tumor development.

In summary, although many complicated questions have not been answered, our study was the first to identify the mTOR/HIF1α/ENO1 pathway in thyroid carcinoma progression and ENO1 as a regulator of CST1, thereby paving the way for early diagnosis and efficient therapy of thyroid carcinoma.

## Data Availability Statement

The data presented in the study are deposited in the NCBI Short Read Archive (SRA) repository, accession number is SUB9428898, and the BioProject ID is PRJNA719990.

## Ethics Statement

The studies involving human participants were reviewed and approved by the Ethics Committee of National Cancer Center/National Clinical Research Center for Cancer/Cancer Hospital, Chinese Academy of Medical Sciences and Peking Union Medical College. The patients/participants provided their written informed consent to participate in this study. The animal study was reviewed and approved by the Ethics Committee of National Cancer Center/National Clinical Research Center for Cancer/Cancer Hospital, Chinese Academy of Medical Sciences and Peking Union Medical College.

## Author Contributions

SL, JL, and YL designed the original study. YL performed the most experiments. LL performed the *in vivo* assay. CA assisted with statistical analysis. XW, ZL, and ZX collected the clinical samples. YL wrote and revised the manuscript draft. All authors read and approved the final manuscript.

## Conflict of Interest

The authors declare that the research was conducted in the absence of any commercial or financial relationships that could be construed as a potential conflict of interest.

## Footnotes

1 targetscan.org

2 http://starbase.sysu.edu.cn/
