# Supplementary material for: α-Enolase Lies Downstream of mTOR/HIF1α and Promotes Thyroid Carcinoma Progression by Regulating CST1
Source: Front Cell Dev Biol. 2021 Apr 21;9:670019. doi: 10.3389/fcell.2021.670019 (PMC8097056; doi:10.3389/fcell.2021.670019)
Supplement: Supplementary file 1 [file Table_1.docx]

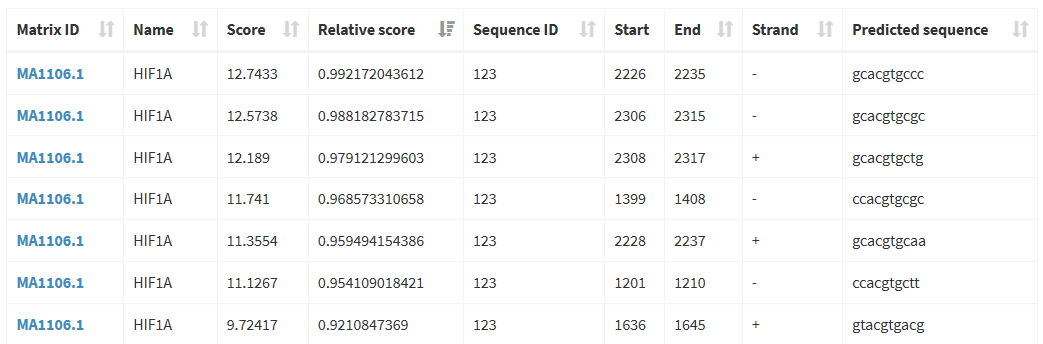


ENO1 promoter

cctgcccaccaccaataaaacttctcaacaagcttacagcaagtgagaaataataactaaatgcaatgccgttgtgctgactacaagcaaatgtgaaccccacagcctgctaacaaagcctaaggagctatggaaacagggagggcatttggcagcctgtgaattttgaaaaagatttcaggacatctctagtaatcattaacattttagctacaatgtgaggtcgaagtgctggctcagaatgtggcgctgcagcctccaagaatcttgcaacggcagcagtccgggatcatgttcggcctctgatttcctgctagatttactggttctggccacagcagagtatctttatctctctaaccatttttatgtaaaataagattgtttggagccctttcagggccacgccttagaattgctctgagagtaccaagtgcctgatttcttagagtccttaccagtaggagatgttggctgttgatttagctgctgagtcatggggctggggatagtggttttcagtagccaaaaacgttagtgtgttgctggtgaaatcacaaactctgcttgcaaaattttactctttttttttttctttttgctgaatttaatcctaaagttcttgcttcttactcactggaaagcaaatactatggtctcaataggccttaatttttaaccaacgccatggaaacctgtctgtagctaagaggataaaaatgctaggggttaaggaagaaaagcaggttttgcagctgaccagaagaaataattaaattcaagtacatatttgaacacctctttacaaggaaatgaggaaaatcagatgttttagttctcttacagaccagcagtaatgttgaaaaatataaaagtagccgtaacagaaaacgaagtgatcatctcagccctcccttggagtggggggatatattgcggagctgagttttaatggctatcagttcttgatcagaaaagtgaaatcactccaactccttccgtattccacggaatatgacccgtcttgaactttgagaaattccccgggtctgtgtgaaattccagttccaaataattcacatgaatggttcacaagaatccaggtaggtttgatctagttccctagatgggaatgagagggatgtaaaatttgagaggcaacatgacatggactagagagagaatgaattaacactggggaagcacgtgggggcgcgccggacacctagaaggcactcaaaaagtatgaggtagaattaaaggaaagtgaaccttcctttggctgtctcggcctgattctcaccgcagcaaggagaaagggacagggtcaccttggcgggtgccaggcggagcccggccagacgggcgcggctggagggcagcgcgcaggcgcaggcggcgcacgtggccccggacacgcagcgctgcggcggggccagcgccccaaccccggagtggggccgaggcgcgcagcctctcaacgactcgacgccaacggggtctctcggatcgcggcgcgaggcaggaggggcggggcaggagggccgcggcaggcccgggccgccaaagttgtcagcaaggtcgagggccggacgtggggccccagagcgacgctgagtgcgtgcgggactcggagtacgtgacggagccccgagctctcatgcccgccacgccgccccgggccatcccccggagccccggctccgcacaccccagttcggctcaccggtcctatctggggccagagtttcgcccgcaccactacagggccgctggggagtcggggccccccagatctgcccgcctcaagtccgcgggacgtcacccccctttccacgctactgcagccgtcgcagtcccacccctttccgggaggtgagggaatgagtgacggctctcccgacgaatggcgaggcggagctgagggggcgtgccccggaggcgggaagtgggtggggctcgccttagctaggcaggaagtcggcgcgggcggcgcggacagtatctgtgggtacccggagcacggagatctcgccggctttacgttcacctcggtgtctgcagcaccctccgcttcctctcctaggcgacgagacccagtggctaggtaatgattggggaagggctgagcaacgggcaggcggcgcagggcttttctgggcttgtggggctcttttccctccgagggaggcggcgcgcccgcaggcccagcgtggcggggtcgggggcacgtgcaagccgcctccctgggtgggaactggcgcccccgctcaccccgaggcccggggcgggggcgcaatgccaggcgcacgtgctgggcccccgatttctttggaggggaaagtgcagatcaacacgtgattaccgactgcggggccgagtcactgtcagggaaggtcgggagctgcacacgcgagctgcttagcacagcgggagttgggagtggaagttgcttttccatcgtagccaagtgtaaggtttttgggatagacgcagactt
