# Supplementary material for: α-Enolase Lies Downstream of mTOR/HIF1α and Promotes Thyroid Carcinoma Progression by Regulating CST1
Source: Front Cell Dev Biol. 2021 Apr 21;9:670019. doi: 10.3389/fcell.2021.670019 (PMC8097056; doi:10.3389/fcell.2021.670019)
Supplement: Supplementary file 2 [file Table_2.DOCX]

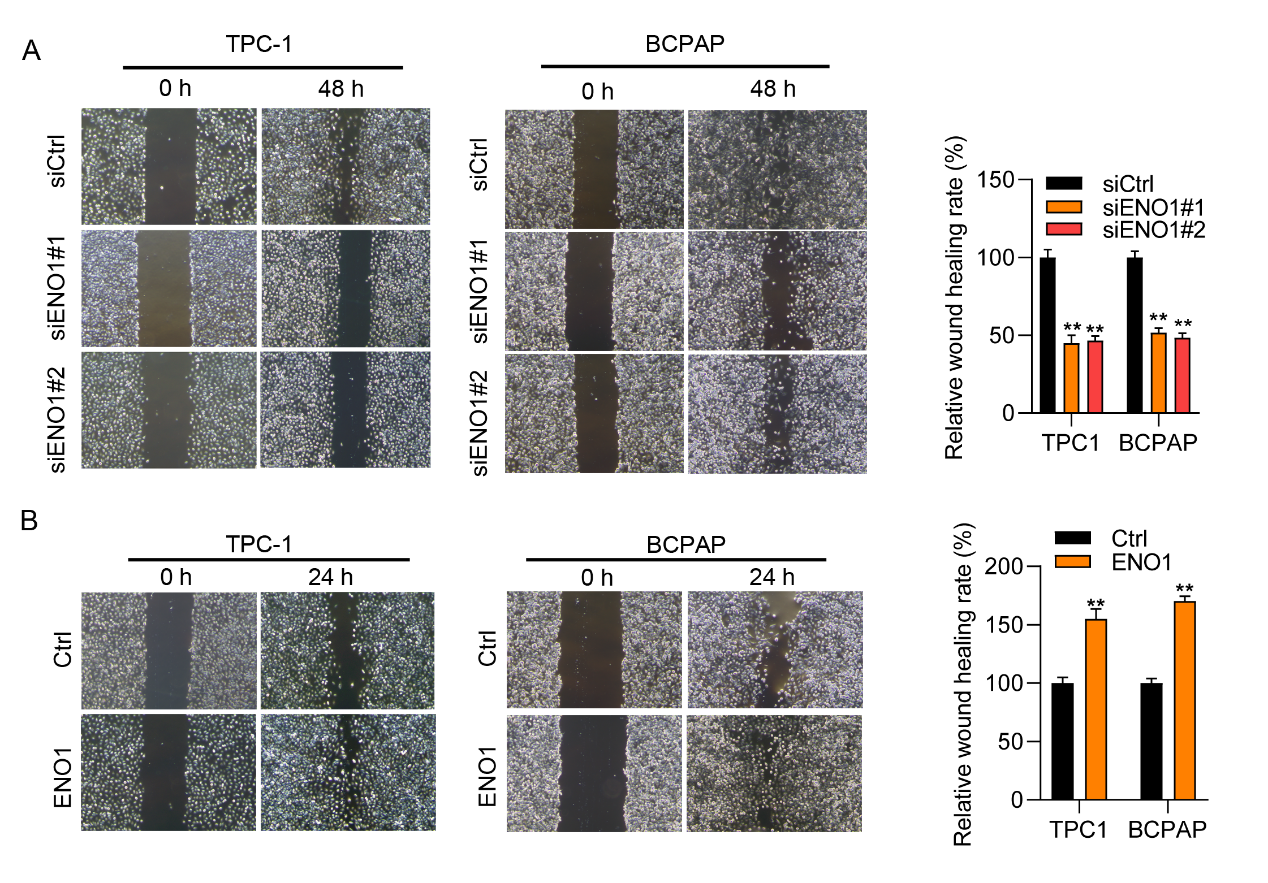


**Figure S1. ENO1 regulates the migration of thyroid cancer cells.**

(A) Migration capabilities of TPC1 and BCPAP cells knockdown ENO1 were evaluated by wound healing assay. Images and quantitation are shown.

(B) Migration capabilities of TPC1 and BCPAP cells overexpression ENO1 were evaluated by wound healing assay. Images and quantitation are shown.


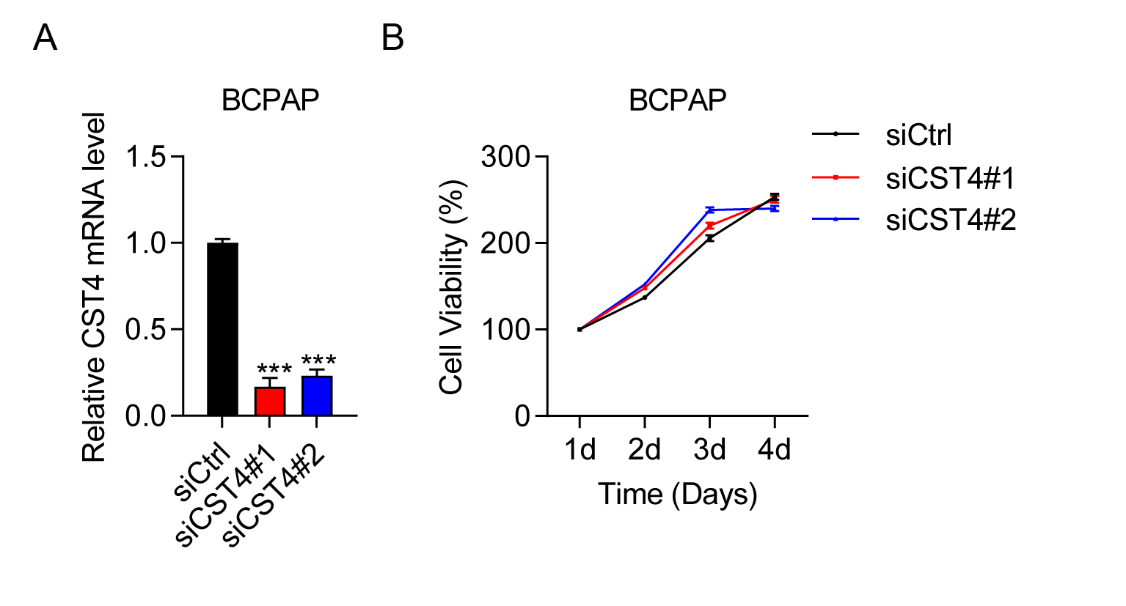


**Figure S2. Downregulation of CST4 has no effect on cell proliferation of BCPAP cells.**

(A) qPCR analysis of CST4 knockdown efficiency in BCPAP cells transfected with siCtrl, siCST4#1, and siCST4#2.

(B) CCK8 analysis of cell proliferation in BCPAP cells transfected with siCtrl, siCST4#1, and siCST4#2.
